# Repurposing existing products to accelerate injury recovery (REPAIR) of military relevant musculoskeletal conditions

**DOI:** 10.3389/fbioe.2022.1105599

**Published:** 2023-01-09

**Authors:** Andrew R. Clark, Timothy C Mauntel, Stephen M Goldman, Christopher L. Dearth

**Affiliations:** ^1^ Research and Surveillance Division, DoD-VA Extremity Trauma and Amputation Center of Excellence, Bethesda, MD, United States; ^2^ Department of Surgery, Uniformed Services University of the Health Sciences—Walter Reed National Military Medical Center, Bethesda, MD, United States; ^3^ Womack Army Medical Center, Fort Bragg, NC, United States

**Keywords:** military personnel, musculoskeletal diseases, trauma, wound healing, drug development

## Abstract

Musculoskeletal injuries (MSKIs) are a great hindrance to the readiness of the United States Armed Forces through lost duty time and reduced operational capabilities. While most musculoskeletal injuries result in return-to-duty/activity with no (functional) limitations, the healing process is often long. Long healing times coupled with the high frequency of musculoskeletal injuries make them a primary cause of lost/limited duty days. Thus, there exists an urgent, clinically unmet need for interventions to expedite tissue healing kinetics following musculoskeletal injuries to lessen their impact on military readiness and society as a whole. There exist several treatments with regulatory approval for other indications that have pro-regenerative/healing properties, but few have an approved indication for treating musculoskeletal injuries. With the immediate need for treatment options for musculoskeletal injuries, we propose a paradigm of Repurposing Existing Products to Accelerate Injury Recovery (REPAIR). Developing treatments *via* repurposing existing therapeutics for other indications has shown monumental advantages in both cost effectiveness and reduced time to bring to market compared to novel candidates. Thus, undertaking the needed research efforts to evaluate the effectiveness of promising REPAIR-themed candidates has the potential to enable near-term solutions for optimizing musculoskeletal injuries recovery, thereby addressing a top priority within the United States. Armed Forces. Herein, the REPAIR paradigm is presented, including example targets of opportunity as well as practical considerations for potential technical solutions for the translation of existing therapeutics into clinical practice for musculoskeletal injuries.

## 1 Introduction

Combat- and non-combat related musculoskeletal injuries (MSKIs) are major causes of lost duty days within the United States Armed Forces. The amount of MSKI-related medically-restricted duty time is a significant impediment to the medical readiness of the United States Armed Forces. While combat-related MSKIs are often more severe, non-combat-related musculoskeletal injuries are more common and account for the majority of the lost duty time (Schneider et al., 2000). MSKIs affect approximately 500,000 Service members annually (Armed Forces Health Surveillance Branch, 2020), and account for 65% of the medical non-deployable population (Molloy et al., 2020). The total economic impact of musculoskeletal injuries within the United States Armed Forces is estimated at $3.7 billion per year, of which direct medical related costs exceed $700 million (Nindl et al., 2013). MSKIs are an even larger burden among United States civilians, with an estimated 1 in 2 adults afflicted with a musculoskeletal disorder, burdening society to the tune of $874 billion annually (United States Bone and Joint Initiative, 2016).

Owing to the body’s endogenous capacity to repair some damaged tissues, the majority of MSKIs result in eventual return-to-duty/activity with no long-term limitations. This is especially true for those on the minor end of the severity spectrum. As such, traditional non-surgical treatment approaches for MSKIs focus on pain management, immobilization, managing inflammation, and physical rehabilitation (Wascher and Bulthuis, 2014). Even with these treatment approaches, recovery is often a long process. For the most common MSKIs, recovery times result in more than 14 limited duty days per injury, with some resulting in 120 limited duty days (Ruscio et al., 2010). During this time, physical activity is commonly limited in a manner that reduces Service member’s function and their ability to perform required duties. As such, there exists an urgent clinically unmet need to expedite tissue healing kinetics following MSKI. Stated differently, the ability to accelerate MSKI healing rate would greatly benefit military and civilian populations alike.

The pressing need to accelerate the endogenous MSKSI recovery timelines requires the development and evaluation of novel therapeutic agents. Traditionally, this type of development lifecycle would require an extensive investment of time and resources into the research and development pipeline needed to develop novel therapeutics (e.g., small molecules and/or orthobiologics) *de novo*. However, given the current existence of a broad portfolio of interventions already under regulatory approval for other indications, there potentially exists an opportunity to leverage or repurpose existing clinically available agents for the accelerated healing of MSKIs to enable near-term, clinically meaningful improvements in the current standard of care, subsequent MSKI outcomes, and readiness of the United States Armed Forces. Notably, historic efforts which utilize a repurposed intervention are up to 10 times less expensive to bring to market and do so in about half the amount of time, compared to novel interventions (Pushpakom et al., 2019). Thus, Repurposing Existing Products to Accelerate Injury Recovery (REPAIR) may be the ideal paradigm for making near-term progress for decreasing the burden of MSKIs within the United States Armed Forces. To that end, the intent of this perspective is to provide a theoretical framework for the types of MSKIs that could be addressed, potential targets of opportunity for further research, and practical considerations for REPAIR.

## 2 Types of musculoskeletal injuries

In the United States Armed Forces, the most common acute MSKIs are sprains/strains (48.5%), contusion/superficial injuries (13.9%), and fractures (10.5%) (Stahlman and Taubman, 2018). Acute MSKIs occur when mechanical properties of a tissue are exceeded and tissue rupture occurs. This can occur due to forces generated within the body (e.g., non-contact anterior cruciate ligament sprain) or from an outside traumatic force (e.g., laceration). Acute MSKIs follow the typical four-phased wound healing process of hemostasis, inflammation, proliferation, and remodeling (Velnar et al., 2009). Dysfunction in the endogenous repair process or repeated re-injury before repair is complete can cause acute MSKIs to become chronic and may result in them adopting pathophysiological characteristics of overuse MSKIs (Arendt-Nielsen et al., 2011).

Overuse MSKIs are four times more common than acute MSKIs in Service members and most commonly include stress fractures, tendinitis, bursitis, and fasciitis (Hauret et al., 2010). These injuries are caused by repeated physical activity that exceeds the body’s ability to adapt. This may be due to normal tissue repair being insufficient for the rate of tissue breakdown or from dysfunction of the tissue repair process itself (Aicale et al., 2018). Regardless, insufficient tissue repair results in degeneration of musculoskeletal tissues, causing inflammation and pain.

Acute and overuse MSKIs present unique challenges that likely require different treatment modalities (Walsh et al., 2008; Dhillon et al., 2017). A successful MSKI therapeutic (or combination thereof) will likely need to be tailored to both the affected tissues and the cause of injury. Not only do tissues contain unique cell populations that respond differently to specific signals, but their function and organization also vastly differ. Histologically, tissues can have drastic differences such as a tissue that provides passive structure and is mostly extracellular matrix (e.g., bone), or a tissue that is highly cellular whose major function is movement (e.g., skeletal muscle) (Betts et al., 2013). There are differences in other fundamental traits, such as vascularity—e.g., cartilage is avascular and skeletal muscle is highly vascular (Betts et al., 2013).

To this end, performing research using the REPAIR paradigm has the potential to identify therapeutics that target endogenous musculoskeletal recovery mechanisms. By developing these candidates for specific MSKI indications, they can aid in the faster healing of injured tissue and a more timely return of function to pre-injury levels. Doing so would culminate in earlier return-to-duty/activity and mitigate re-injury risks, further reducing long term loss of duty.

## 3 Examples of targets of opportunity

The following sections provide high-level overviews of some physiologic targets of opportunity for potential REPAIR candidates which may warrant consideration for further research investigations. Information is summarized in Table 1.

**TABLE 1:** Examples of existing therapeutics for potential REPAIR utilization.

Modality	Clinically available therapeutics	Relevant tissues	Impact on tissues	Relevance to MSKI
Anabolic pathways	• Testosterone (androgenic-anabolic steroids) • Mecasermin • Somatropin • Abaloparatide • Teriparatide	• Bone • Cartilage • Muscle • Ligament • Tendon	• Increase bone mineralization density Kenny et al. (2010) • Muscle fiber hypertrophy Yoshida and Delafontaine. (2020), Urban et al. (1995); Kenny et al. (2010) • Tenocyte proliferation Disser et al. (2019) • Increase collagen production and tensile strength Provenzano et al. (2007) • Chondrocyte proliferation and differentiation Dixit et al. (2021) • Osteoblast proliferation and differentiation Tahimic et al. (2013), Wang et al. (2007) • Prevent apoptosis Wang et al. (2007)	• Increase bone mineralization • Mitigate atrophy • Increase tensile strength • Increase tissue mass
Perfusion	• Iloprost • Losartan • Nitroglycerin • Sildenafil • Tadalafil	• Bone • Cartilage • Muscle • Tendon	• Vasodilation/anti-hypertensive and increase blood flow Abrams. (1985); Steinberg et al. (1988); Halcox et al. (2002); Rajkumar et al. (2013) • Suppress osteoclast mediated bone resorption Chen et al. (2015) • Decrease fibrosis Garg et al. (2014); Utsunomiya et al. (2019) • Restore injured tendon biomechanical strength Yuan et al. (2003) • Increase bone mass Kim et al. (2020)	• Improve fracture healing • Decrease fibrotic tissue • Increase bone formation • Improve recovery from tendinosis • Mitigate atrophy • Increase bone formation
BMP pathway	• Dibotermin alfa (rhBMP2) • Lovastatin • Simvastatin • Tacrolimus • Denosumab	• Bone	• Enhance osteogenesis Song et al. (2003); Gutierrez et al. (2008); Wu et al. (2016); Sangadala et al. (2019) • Inhibits osteoclastogenesis Gerstenfeld et al. (2009)	• Increase bone formation
Other	• Erythropoietin	• Bone • Cartilage • Muscle • Tendon	• Anti-apoptotic Ghezzi and Brines. (2004) • Stimulate hematopoesis Suresh et al. (2020) • Increase vascularization Suresh et al. (2020)	• Mitigate atrophy • Support tissue growth
	• PGC1α agonists (pioglitazone)	• Cartilage • Muscle	• Aid chondrogenesis Kawakami et al. (2005) • Decrease chondrocyte catabolism Zhao et al. (2014) • Increase mitochondria biogenesis Liang and Ward. (2006) • Increase ROS scavenger production Baldelli et al. (2014) • Correct oxidative pathophysiology Southern et al. (2019)	• Mitigate atrophy • Correct dysfunctional metabolism

BMP, bone morphogenic protein; PGC1α, Proliferator-activated Receptor Gamma Coactivator 1-Alpha; REPAIR, repurposing existing products to accelerate injury recovery.

### 3.1 Anabolic pathways

The reconstitution of damaged tissue to restore tissue mechanics, end organ function (e.g., muscle contractility), and limb utility is the crux of MSKI recovery. Substantial anabolic activity is required to synthesize and arrange the biomolecules needed for this rebuilding effort. Thus, the targeted, well-controlled and timed boosting of anabolic pathways may be an opportunity to accelerate tissue repair and shorten MSKI recovery time (Demling, 2009; Song et al., 2013; Roberts and Ke, 2018). Additionally, anabolic agents may be beneficial in preventing secondary atrophy caused by injuries or disuse during the recovery period (Isaacs et al., 2011; Gerber et al., 2015). Existing approved modulators of the anabolic pathways that could be associated with improved MSKI recovery include (but not limited to): insulin-like growth factor 1 (mecasermin)/growth hormone (somatropin) (Provenzano et al., 2007; Tahimic et al., 2013; Disser et al., 2019; Yoshida and Delafontaine, 2020; Dixit et al., 2021), parathyroid hormone (abaloparatide, teriparatide) (Wang et al., 2007; Ellegaard et al., 2010), and testosterone (androgenic-anabolic steroids) (Urban et al., 1995; Kenny et al., 2010; Wu et al., 2014). The ubiquitous effects of anabolic pathways make them likely to be beneficial for many different MSKIs but current products are often associated with adverse effects (Fintini et al., 2009; Albano et al., 2021; Vall and Parmar, 2022). Therefore, development and evaluation of modified products as well as delivery mechanisms, timing, and dosage could help bring effective anabolic treatments for MKSI to fruition.

### 3.2 Perfusion

Blood flow is essential to provide oxygen, nutrients, and cells to tissue while also removing wastes. This is especially important for tissue repair, due to the high amount of cellular activity that occurs during this process. Injuries often hyper-neovascularizatize (i.e., angiogenesis) within the affected tissue during the repair process, which then returns to pre-injury levels over the course of tissue remodeling (Fenwick et al., 2002; Hankenson et al., 2011). Rapid vascularization is likely more important in the initial stages of repair where the tissue might be ischemic due to damaged blood vessels. Pro-angiogenic factors, such as vascular endothelial growth factor (VEGF), can accelerate MSKI healing (Street et al., 2002). However, none of the existing pro-angiogenic factors have been successfully implemented within a clinical setting. Alternatively, therapies that increase perfusion through the endogenous vasculature may confer similar benefits as increased vascularization (MalekiGorji et al., 2020). Existing approved drugs including (but are not limited to) sildenafil, tadalafil, nitroglycerin, epoprostenol, and iloprost have been shown to increase perfusion through vasodilation. Anti-hypertensive medications, such as losartan, cause vasodilation and could prove to be useful as well. These therapeutics have well established clinical safety profiles and thus may serve as potential targets of opportunity for REPAIR-themed research efforts to evaluate their efficacy within the context of MSKIs as means to facilitate the supply of oxygen and nutrients to the damaged tissue.

### 3.3 Bone morphogenic protein pathway

Bone morphogenic proteins (BMPs) are a family of potent osteogenic activators that have been shown to facilitate *de novo* bone formation in both pre-clinical and clinical applications (Boyne et al., 2005; Hashimoto et al., 2020). While BMPs induce potent osteogenic activity, their delivery needs to be tightly controlled due to their ability to cause ectopic bone formation and other adverse side effects (James et al., 2016). Small molecule drugs that stimulate BMP pathways might also aid in bone regeneration. Existing approved agents that activate BMP pathways include (but are not limited to) tacrolimus (immunosuppressant) (Sangadala et al., 2019), lovastatin (statin) (Gutierrez et al., 2008), and simvastatin (statin) (Song et al., 2003). Investment in REPAIR-themed research efforts within this context could yield the development of a short-term localized administration (i.e., an injection) of a BMP-activator to a fracture site with the goal of hastening bone formation at the injury site and thus allow for return to normal weight bearing activity earlier than would otherwise be possible with the body’s endogenous healing processes alone.

### 3.4 Other targets of opportunity

There exist other therapeutic candidates with different mechanisms that may prove beneficial for treating MSKIs. For example a receptor activator of nuclear factor kappa-Β ligand (RANKL) inhibitor, Denosumab, can act as a potent osteoclastogenesis inhibitor and may increase bone formation following an MSKI (Hanley et al., 2012). The emerging field of metabolic reprogramming may yield beneficial therapeutics for MSKIs. In addition to altering energy production, studies show metabolic reprogramming interventions can modulate inflammation and control stem cell differentiation (Shyh-Chang and Ng, 2017; Palsson-McDermott and O'Neill, 2020). The role of metabolism in the pathophysiology of most MSKIs requires additional work to be fully understood. However, one metabolic reprogramming approach that has shown promise is activating the pathway of peroxisome proliferator-activated receptor gamma coactivator 1-alpha (PGC1α), a regulator of energy metabolism and mitochondria biogenesis, for the recovery of skeletal muscle injuries (Liang and Ward, 2006; Southern et al., 2019). Pioglitazone is an FDA approved drugs that can activate PGC1α and have beneficial anti-inflammatory effects (Komen and Thorburn, 2014). Looking into therapeutics that work through multimodal mechanisms related to recovery could prove to be more effective than a therapeutic targeting a single mechanism. Erythropoietin (epoetin alfa) is a multimodal therapeutic that stimulates red blood cells and has anti-apoptotic effects on a variety of tissues (Ghezzi and Brines, 2004). Thus, erythropoietin may enhance oxygenation, decrease secondary injury, and aid in tissue repair for a variety of MSKIs.

While the sections above (and within Table 1) details some potential candidates that deserve further research for their applicability in MSKIs, it is important to note that this is not an exhaustive list and as such there exists many other potential candidates with known, relevant biologic effects which should be considered for REPAIR-themed investigations. Furthermore, in addition to evaluating therapeutics with known REPAIR-relevant mechanisms, high throughput screening of existing/approved drug libraries would likely be beneficial to identify additional candidates that may be efficacious for treating MSKIs.

## 4 Practical considerations for repurposing existing products to accelerate injury recovery solutions

There are several important design criteria for scientists and clinicians to consider when evaluating potential REPAIR-themed candidates (Table 2). As stated previously, a majority of MSKIs eventually recover on their own and Service members return-to-duty/activity with little/no limitations. As such, adverse side effects from a therapeutic should be as minimal as possible (if at all), as to not exceed the therapies’ expected benefit(s). Any developed REPAIR therapeutic would ideally be additive or synergistic with current treatments of rehabilitation, surgery, and pain management. To meet the high demand imposed by MSKIs, a treatment would need to be mass produced and cost effective. The complexity of the therapeutic should be kept low. Ideally, a REPAIR therapeutic can be self-administered. If increasing the dose at the site of injury while minimizing off target effects is needed, localized approaches such as a topical cream or patch, or at a higher complexity, an injection, might be useful. A REPAIR treatment that requires a medical professional to administer is best if limited to the need of a single administration to avoid repeated visits. An ideal effective treatment would be kept in stock at medical facilities and perhaps even carried by a field medic and thus a long shelf-life and ease of storage, small size, and stable across a range of temperatures, are desired.

**TABLE 2:** Suggested design criteria for REPAIR solutions.

**Design criteria**	**Rationale**	**Suggested criteria**
Minimal severity and prevalence of adverse effects	Adverse effects from a therapeutic should not exceed the benefit received from reducing MSKI recovery time	Effective dose << maximum tolerable dose, No adverse effects
Restore function to injured tissue	Allow Service member to perform duties without restriction	Reduced time to return to duty/activity
Effective as part of a total treatment regimen	Additive or synergistic with current treatments of rehabilitation, surgery, and pain management	REPAIR solution + current standard of care > standard of care
Decrease future MSKI risk	Prevent lost duty from future MSKIs	Secondary MSKI risk ≤ primary MSKI risk
Low cost	Complex and high-cost therapeutics can eliminate the benefit of a MSKI therapeutic	Treatment cost < cost for lost duty
Simple administration	Reduce administration errors/decrease cost of medical professional time	Self-delivered, 1 time delivery by medical professional at time of diagnosis
Long shelf-life	Facilities should be able to maintain a stock for short diagnosis to treatment time	1 year+

MSKI, musculoskeletal injury; REPAIR, repurposing existing products to accelerate injury recovery.

In addition to the efficacy of a REPAIR therapeutic accelerating MSKI recovery time, consideration should also be taken for long (er)-term implications. MSKIs can increase the risk for a subsequent MSKI by seven times (Schneider et al., 2000). A specific example is the risk of developing osteoarthritis, the leading cause of disability discharge (Cameron et al., 2016), is greatly increased by a joint MSKI (Buckwalter and Brown, 2004; Wang et al., 2020). Thus, mitigating secondary and tertiary injury risks *via* improved recovery could yield long-term benefits for lost duty by the reduction in total injury occurrence. Research using risk factors for future injuries as an outcome metric would be beneficial in addition to metrics related to regain of function from the current injury.

## 5 Conclusion

MSKIs result in a significant burden on the United States Armed Forces in terms of both costs and lost duty time, and thus are a major impediment to readiness (Schneider et al., 2000; Nindl et al., 2013)^,^. There is an unmet need for therapies that accelerate MSKI recovery time and mitigate MSKI recurrence risks. There are existing therapies for other indications that, pending beneficial efficacy data from needed research investigations, may fill this need. Notably, if/when forthcoming research data support its use, rather than the monumental investment of resources (i.e., time and money) needed to develop and evaluate an intervention *de novo*, the REPAIR paradigm described herein has the benefit of leveraging prior investments, often from the federal sector (e.g., National Institute of Health, Department of Defense), such that research and design of potential MSKI specific indications would occur in a more cost and time effective manner. Thus, utilizing the REPAIR paradigm to leverage existing technologies for the development of treatments for common MSKIs could make more significant near-term progress at decreasing the present MSKI burden and increasing overall military readiness.

## Data Availability

The raw data supporting the conclusion of this article will be made available by the authors, without undue reservation.

## References

[B1] Abrams J. (1985). Hemodynamic effects of nitroglycerin and long-acting nitrates. Am. Heart J. 110, 217–224. 10.1016/0002-8703(85)90490-9 3925741

[B2] Aicale R. Tarantino D. Maffulli N. (2018). Overuse injuries in sport: A comprehensive overview. J. Orthop. Surg. Res. 13 (1), 309. 10.1186/s13018-018-1017-5 30518382 PMC6282309

[B3] Albano G. D. Amico F. Cocimano G. Liberto A. Maglietta F. Esposito M. (2021). Adverse effects of anabolic-androgenic steroids: A literature review. Healthc. (Basel) 9 (1), 97. 10.3390/healthcare9010097 PMC7832337 33477800

[B4] Arendt-Nielsen L. Fernandez-de-Las-Penas C. Graven-Nielsen T. (2011). Basic aspects of musculoskeletal pain: From acute to chronic pain. J. Man. Manip. Ther. 19 (4), 186–193. 10.1179/106698111X13129729551903 23115471 PMC3201649

[B5] Armed Forces Health Surveillance Branch (2020). Absolute and relative morbidity burdens attributable to various illnesses and injuries, active component, U.S. Armed Forces, 2019. MSMR 27 (5), 2–9. 32479095

[B6] Baldelli S. Aquilano K. Ciriolo M. R. (2014). PGC-1α buffers ROS-mediated removal of mitochondria during myogenesis. Cell Death Dis. 5 (11), e1515. 10.1038/cddis.2014.458 25375380 PMC4260723

[B7] Betts J. G. Desaix P. Johnson E. Johnson J. E. Korol O. Kruse D. (2013). Anatomy & physiology. Houston, TX: Openstax.

[B8] Boyne P. J. Lilly L. C. Marx R. E. Moy P. K. Nevins M. Spagnoli D. B. (2005). De novo bone induction by recombinant human bone morphogenetic protein-2 (rhBMP-2) in maxillary sinus floor augmentation. J. Oral Maxillofac. Surg. 63 (12), 1693–1707. 10.1016/j.joms.2005.08.018 16297689

[B9] Buckwalter J. A. Brown T. D. (2004). Joint injury, repair, and remodeling: Roles in post-traumatic osteoarthritis. Clin. Orthop. Relat. Res. 423, 7–16. 10.1097/01.blo.0000131638.81519.de 15232420

[B10] Cameron K. L. Driban J. B. Svoboda S. J. (2016). Osteoarthritis and the tactical athlete: A systematic review. J. Athl. Train. 51 (11), 952–961. 10.4085/1062-6050-51.5.03 27115044 PMC5224737

[B11] Chen S. Grover M. Sibai T. Black J. Rianon N. Rajagopal A. (2015). Losartan increases bone mass and accelerates chondrocyte hypertrophy in developing skeleton. Mol. Genet. Metab. 115 (1), 53–60. 10.1016/j.ymgme.2015.02.006 25779879 PMC4426054

[B12] Demling R. H. (2009). Nutrition, anabolism, and the wound healing process: An overview. Eplasty 9, e9. 19274069 PMC2642618

[B13] Dhillon H. Dhillon S. Dhillon M. S. (2017). Current concepts in sports injury rehabilitation. Indian J. Orthop. 51 (5), 529–536. 10.4103/ortho.IJOrtho_226_17 28966376 PMC5609374

[B14] Disser N. P. Sugg K. B. Talarek J. R. Sarver D. C. Rourke B. J. Mendias C. L. (2019). Insulin-like growth factor 1 signaling in tenocytes is required for adult tendon growth. FASEB J. 33 (11), 12680–12695. 10.1096/fj.201901503R 31536390 PMC6902672

[B15] Dixit M. Poudel S. B. Yakar S. (2021). Effects of GH/IGF axis on bone and cartilage. Mol. Cell Endocrinol. 519, 111052. 10.1016/j.mce.2020.111052 33068640 PMC7736189

[B16] Ellegaard M. Jørgensen N. R. Schwarz P. (2010). Parathyroid hormone and bone healing. Calcif. Tissue Int. 87 (1), 1–13. 10.1007/s00223-010-9360-5 20428858

[B17] Fenwick S. A. Hazleman B. L. Riley G. P. (2002). The vasculature and its role in the damaged and healing tendon. Arthritis Res. 4 (4), 252–260. 10.1186/ar416 12106496 PMC128932

[B18] Fintini D. Brufani C. Cappa M. (2009). Profile of mecasermin for the long-term treatment of growth failure in children and adolescents with severe primary IGF-1 deficiency. Ther. Clin. Risk Manag. 5 (3), 553–559. 10.2147/tcrm.s6178 19707272 PMC2724186

[B19] Garg K. Corona B. T. Walters T. J. (2014). Losartan administration reduces fibrosis but hinders functional recovery after volumetric muscle loss injury. J. Appl. Physiol(1985) 117 (10), 1120–1131. 10.1152/japplphysiol.00689.2014 25257876

[B20] Gerber C. Meyer D. C. Fluck M. Benn M. C. von Rechenberg B. Wieser K. (2015). Anabolic steroids reduce muscle degeneration associated with rotator cuff tendon release in sheep. Am. J. Sports Med. 43 (10), 2393–2400. 10.1177/0363546515596411 26304962

[B21] Gerstenfeld L. C. Sacks D. J. Pelis M. Mason Z. D. Graves D. T. Barrero M. (2009). Comparison of effects of the bisphosphonate alendronate versus the RANKL inhibitor denosumab on murine fracture healing. J. Bone Min. Res. 24 (2), 196–208. 10.1359/jbmr.081113 PMC6961532 19016594

[B22] Ghezzi P. Brines M. (2004). Erythropoietin as an antiapoptotic, tissue-protective cytokine. Cell Death Differ. 11 Suppl 1, S37–S44. 10.1038/sj.cdd.4401450 15243580

[B23] Gutierrez G. E. Edwards J. R. Garrett I. R. Nyman J. S. McCluskey B. Rossini G. (2008). Transdermal lovastatin enhances fracture repair in rats. J. Bone Min. Res. 23 (11), 1722–1730. 10.1359/jbmr.080603 PMC2685484 18597639

[B24] Halcox J. P. Nour K. R. Zalos G. Mincemoyer R. A. Waclawiw M. Rivera C. E. (2002). The effect of sildenafil on human vascular function, platelet activation, and myocardial ischemia. J. Am. Coll. Cardiol. 40 (7), 1232–1240. 10.1016/s0735-1097(02)02139-3 12383570

[B25] Hankenson K. D. Dishowitz M. Gray C. Schenker M. (2011). Angiogenesis in bone regeneration. Injury 42 (6), 556–561. 10.1016/j.injury.2011.03.035 21489534 PMC3105195

[B26] Hanley D. A. Adachi J. D. Bell A. Brown V. (2012). Denosumab: Mechanism of action and clinical outcomes. Int. J. Clin. Pract. 66 (12), 1139–1146. 10.1111/ijcp.12022 22967310 PMC3549483

[B27] Hashimoto K. Kaito T. Furuya M. Seno S. Okuzaki D. Kikuta J. (2020). *In vivo* dynamic analysis of BMP-2-induced ectopic bone formation. Sci. Rep. 10 (1), 4751. 10.1038/s41598-020-61825-2 32179857 PMC7076033

[B28] Hauret K. G. Jones B. H. Bullock S. H. Canham-Chervak M. Canada S. (2010). Musculoskeletal injuries. Am. J. Prev. Med. 38, S61–S70. 10.1016/j.amepre.2009.10.021 20117601

[B29] Isaacs J. Loveland K. Mallu S. Adams S. Wodicka R. (2011). The use of anabolic steroids as a strategy in reversing denervation atrophy after delayed nerve repair. Hand (N Y) 6 (2), 142–148. 10.1007/s11552-011-9331-y 22654697 PMC3092896

[B30] James A. W. LaChaud G. Shen J. Asatrian G. Nguyen V. Zhang X. (2016). A review of the clinical side effects of bone morphogenetic protein-2. Tissue Eng. Part B Rev. 22 (4), 284–297. 10.1089/ten.TEB.2015.0357 26857241 PMC4964756

[B31] Kawakami Y. Tsuda M. Takahashi S. Taniguchi N. Esteban C. R. Zemmyo M. (2005). Transcriptional coactivator PGC-1α regulates chondrogenesis via association with Sox9. Proc. Natl. Acad. Sci. U. S. A. 102 (7), 2414–2419. 10.1073/pnas.0407510102 15699338 PMC548985

[B32] Kenny A. M. Kleppinger A. Annis K. Rathier M. Browner B. Judge J. O. (2010). Effects of transdermal testosterone on bone and muscle in older men with low bioavailable testosterone levels, low bone mass, and physical frailty. J. Am. Geriatr. Soc. 58 (6), 1134–1143. 10.1111/j.1532-5415.2010.02865.x 20722847 PMC3014265

[B33] Kim S.-M. Taneja C. Perez-Pena H. Ryu V. Gumerova A. Li W. (2020). Repurposing erectile dysfunction drugs tadalafil and vardenafil to increase bone mass. Proc. Natl. Acad. Sci. 117 (25), 14386–14394. 10.1073/pnas.2000950117 32513693 PMC7321982

[B34] Komen J. C. Thorburn D. R. (2014). Turn up the power - pharmacological activation of mitochondrial biogenesis in mouse models. Br. J. Pharmacol. 171 (8), 1818–1836. 10.1111/bph.12413 24102298 PMC3976607

[B35] Liang H. Ward W. F. (2006). PGC-1α: A key regulator of energy metabolism. Adv. Physiol. Educ. 30 (4), 145–151. 10.1152/advan.00052.2006 17108241

[B36] MalekiGorji M. Golestaneh A. Razavi S. M. (2020). The effect of two phosphodiesterase inhibitors on bone healing in mandibular fractures (animal study in rats). J. Korean Assoc. Oral Maxillofac. Surg. 46 (4), 258–265. 10.5125/jkaoms.2020.46.4.258 32855373 PMC7469969

[B37] Molloy J. M. Pendergrass T. L. Lee I. E. Chervak M. C. Hauret K. G. Rhon D. I. (2020). Musculoskeletal injuries and United States army readiness Part I: Overview of injuries and their strategic impact. Mil. Med. 185 (9-10), e1461–e1471. 10.1093/milmed/usaa027 32175566

[B38] Nindl B. C. Williams T. J. Deuster P. A. Butler N. L. Jones B. H. (2013). Strategies for optimizing military physical readiness and preventing musculoskeletal injuries in the 21st century. U. S. Army Med. Dep. J., 5–23. 24146239

[B39] Palsson-McDermott E. M. O'Neill L. A. J. (2020). Targeting immunometabolism as an anti-inflammatory strategy. Cell Res. 30 (4), 300–314. 10.1038/s41422-020-0291-z 32132672 PMC7118080

[B40] Provenzano P. P. Alejandro-Osorio A. L. Grorud K. W. Martinez D. A. Vailas A. C. Grindeland R. E. (2007). Systemic administration of IGF-I enhances healing in collagenous extracellular matrices: Evaluation of loaded and unloaded ligaments. BMC Physiol. 7, 2. 10.1186/1472-6793-7-2 17386107 PMC1851714

[B41] Pushpakom S. Iorio F. Eyers P. A. Escott K. J. Hopper S. Wells A. (2019). Drug repurposing: Progress, challenges and recommendations. Nat. Rev. Drug Discov. 18 (1), 41–58. 10.1038/nrd.2018.168 30310233

[B42] Rajkumar D. S. R. Faitelson A. V. Gudyrev O. S. Dubrovin G. M. Pokrovski M. V. Ivanov A. V. (2013). Comparative evaluation of enalapril and losartan in pharmacological correction of experimental osteoporosis and fractures of its background. J. Osteoporos. 2013, 1–5. 10.1155/2013/325693 PMC3562670 23401845

[B43] Roberts S. J. Ke H. Z. (2018). Anabolic strategies to augment bone fracture healing. Curr. Osteoporos. Rep. 16 (3), 289–298. 10.1007/s11914-018-0440-1 29725836 PMC5945805

[B44] Ruscio B. A. Jones B. H. Bullock S. H. Burnham B. R. Canham-Chervak M. Rennix C. P. (2010). A process to identify military injury prevention priorities based on injury type and limited duty days. Am. J. Prev. Med. 38, S19–S33. 10.1016/j.amepre.2009.10.004 20117593

[B45] Sangadala S. Devereaux E. J. Presciutti S. M. Boden S. D. Willet N. J. (2019). FK506 induces ligand-independent activation of the bone morphogenetic protein pathway and osteogenesis. Int. J. Mol. Sci. 20 (8), 1900. 10.3390/ijms20081900 30999619 PMC6515024

[B46] Schneider G. A. Bigelow C. Amoroso P. J. (2000). Evaluating risk of re-injury among 1214 army airborne soldiers using a stratified survival model. Am. J. Prev. Med. 18, 156–163. 10.1016/s0749-3797(99)00177-4 10736552

[B47] Shyh-Chang N. Ng H. H. (2017). The metabolic programming of stem cells. Genes Dev. 31 (4), 336–346. 10.1101/gad.293167.116 28314766 PMC5358754

[B48] Song C. Guo Z. Ma Q. Chen Z. Liu Z. Jia H. (2003). Simvastatin induces osteoblastic differentiation and inhibits adipocytic differentiation in mouse bone marrow stromal cells. Biochem. Biophys. Res. Commun. 308 (3), 458–462. 10.1016/s0006-291x(03)01408-6 12914771

[B49] Song Y. H. Song J. L. Delafontaine P. Godard M. P. (2013). The therapeutic potential of IGF-I in skeletal muscle repair. Trends Endocrinol. Metab. 24 (6), 310–319. 10.1016/j.tem.2013.03.004 23628587 PMC3732824

[B50] Southern W. M. Nichenko A. S. Tehrani K. F. McGranahan M. J. Krishnan L. Qualls A. E. (2019). PGC-1α overexpression partially rescues impaired oxidative and contractile pathophysiology following volumetric muscle loss injury. Sci. Rep. 9 (1), 4079. 10.1038/s41598-019-40606-6 30858541 PMC6411870

[B51] Stahlman S. Taubman S. B. (2018). Incidence of acute injuries, active component. MSMR 25 (7), 2–9. 30047273

[B52] Steinberg H. Medvedev O. S. Luft F. C. Unger T. (1988). Effect of a prostacyclin derivative (iloprost) on regional blood flow, sympathetic nerve activity, and baroreceptor reflex in the conscious rat. J. Cardiovasc Pharmacol. 11 (1), 84–89. 10.1097/00005344-198801000-00013 2450262

[B53] Street J. Bao M. deGuzman L. Bunting S. Peale F. V. Ferrara N. (2002). Vascular endothelial growth factor stimulates bone repair by promoting angiogenesis and bone turnover. Proc. Natl. Acad. Sci. 99 (15), 9656–9661. 10.1073/pnas.152324099 12118119 PMC124965

[B54] Suresh S. Rajvanshi P. K. Noguchi C. T. (2020). The many facets of erythropoietin physiologic and metabolic response. Front. Physiology 10 (1534), 1534. 10.3389/fphys.2019.01534 PMC6984352 32038269

[B55] Tahimic C. G. Wang Y. Bikle D. D. (2013). Anabolic effects of IGF-1 signaling on the skeleton. Front. Endocrinol. (Lausanne) 4, 6. 10.3389/fendo.2013.00006 23382729 PMC3563099

[B56] United States Bone and Joint Initiative (2016). The burden of musculoskeletal Diseases in the United States (BMUS). 3rd Edition. Lombard, IL: United States Bone and Joint Initiative.

[B57] Urban R. J. Bodenburg Y. H. Gilkison C. Foxworth J. Coggan A. R. Wolfe R. R. (1995). Testosterone administration to elderly men increases skeletal muscle strength and protein synthesis. Am. J. Physiol. 269, E820–E826. 10.1152/ajpendo.1995.269.5.E820 7491931

[B58] Utsunomiya H. Gao X. Deng Z. Cheng H. Scibetta A. Ravuri S. (2019). Improvement of cartilage repair with biologically regulated marrow stimulation by blocking TGF-β1 in A rabbit osteochondral defect model. Orthop. J. Sports Med. 7 (7), 2325967119S0026. 10.1177/2325967119S00263 32027515

[B59] Vall H. Parmar M. (2022). Teriparatide. Treasure Island FL: StatPearls. StatPearls Publishing Copyright © 2022, StatPearls Publishing LLC.). 32644674

[B60] Velnar T. Bailey T. Smrkolj V. (2009). The wound healing process: An overview of the cellular and molecular mechanisms. J. Int. Med. Res. 37 (5), 1528–1542. 10.1177/147323000903700531 19930861

[B61] Walsh N. E. Brooks P. Hazes J. M. Walsh R. M. Dreinhöfer K. Woolf A. D. (2008). Standards of care for acute and chronic musculoskeletal pain: The bone and joint decade (2000-2010). Arch. Phys. Med. Rehabil. 89 (9), 1830–1845.e4. 10.1016/j.apmr.2008.04.009 18760171

[B62] Wang L. J. Zeng N. Yan Z. P. Li J. T. Ni G. X. (2020). Post-traumatic osteoarthritis following ACL injury. Arthritis Res. Ther. 22 (1), 57. 10.1186/s13075-020-02156-5 32209130 PMC7092615

[B63] Wang Y. H. Liu Y. Rowe D. W. (2007). Effects of transient PTH on early proliferation, apoptosis, and subsequent differentiation of osteoblast in primary osteoblast cultures. Am. J. Physiol. Endocrinol. Metab. 292 (2), E594–E603. 10.1152/ajpendo.00216.2006 17032929

[B64] Wascher D. C. Bulthuis L. (2014). Extremity trauma: Field management of sports injuries. Curr. Rev. Musculoskelet. Med. 7 (4), 387–393. 10.1007/s12178-014-9242-y 25283054 PMC4596220

[B65] Wu B. W. Berger M. Sum J. C. Hatch G. F. 3rd Schroeder E. T. (2014). Randomized control trial to evaluate the effects of acute testosterone administration in men on muscle mass, strength, and physical function following ACL reconstructive surgery: Rationale, design, methods. BMC Surg. 14, 102. 10.1186/1471-2482-14-102 25481088 PMC4267143

[B66] Wu M. Chen G. Li Y.-P. (2016). TGF-β and BMP signaling in osteoblast, skeletal development, and bone formation, homeostasis and disease. Bone Res. 4 (1), 16009. 10.1038/boneres.2016.9 27563484 PMC4985055

[B67] Yoshida T. Delafontaine P. (2020). Mechanisms of IGF-1-mediated regulation of skeletal muscle hypertrophy and atrophy. Cells 9 (9), 1970. 10.3390/cells9091970 32858949 PMC7564605

[B68] Yuan J. Murrell G. A. Wei A. Q. Appleyard R. C. Del Soldato P. Wang M. X. (2003). Addition of nitric oxide via nitroflurbiprofen enhances the material properties of early healing of young rat Achilles tendons. Inflamm. Res. 52 (6), 230–237. 10.1007/s00011-003-1167-7 12835894

[B69] Zhao X. Petursson F. Viollet B. Lotz M. Terkeltaub R. Liu-Bryan R. (2014). Peroxisome proliferator-activated receptor γ coactivator 1α and FoxO3A mediate chondroprotection by AMP-activated protein kinase. Arthritis Rheumatol. 66 (11), 3073–3082. 10.1002/art.38791 25047750 PMC4343036

